# Thermomechanical Properties and Biodegradation Behavior of Itaconic Anhydride-Grafted PLA/Pecan Nutshell Biocomposites †

**DOI:** 10.3390/polym14245532

**Published:** 2022-12-17

**Authors:** Sarai Agustin-Salazar, Marco Ricciulli, Veronica Ambrogi, Pierfrancesco Cerruti, Gennaro Scarinzi

**Affiliations:** 1 Institute for Polymers, Composites and Biomaterials (IPCB-CNR), Via Campi Flegrei 34, 80078 Pozzuoli, Italy; 2 Department of Chemical and Metallurgical Engineering (DIQyM), University of Sonora, Building 5B, Del Conocimiento, Centro, Hermosillo C.P. 83000, Sonora, Mexico; 3 Department of Chemical, Materials and Production Engineering (DICMAPI), University of Naples Federico II, Piazzale Tecchio 80, 80125 Naples, Italy; 4 Institute for Polymers, Composites and Biomaterials (IPCB-CNR), Via Gaetano Previati, 1/E, 23900 Lecco, Italy

**Keywords:** polylactic acid (PLA), pecan nutshell (PNS), itaconic anhydride (IA), PLA grafting, biocomposites, ball milling, thermal annealing, soil burial, biodegradation

## Abstract

The use of lignocellulose-rich biowaste as reinforcing filler in biodegradable polymers represents a sustainable option to obtain cost-effective bio-based materials to be used for several applications. In addition, the scarce polymer–biofiller interaction can be improved by reactive functionalization of the matrix. However, the obtained biocomposites might show high thermal deformability and possibly a slow biodegradation rate. In this work, polylactic acid (PLA) was first chemically modified with itaconic anhydride, and then biocomposites containing 50 wt.% of pecan (*Carya illinoinensis*) nutshell (PNS) biowaste were prepared and characterized. Their physical and morphological properties were determined, along with their biodegradation behavior in soil. Moreover, the effects of two environmentally friendly physical treatments, namely ball-milling of the filler and thermal annealing on biocomposites, were assessed. Grafting increased PLA thermal-oxidative stability and crystallinity. The latter was further enhanced by the presence of PNS, achieving a 30% overall increase compared to the plain matrix. Accordingly, the biocomposites displayed mechanical properties comparable to those of the plain matrix. Thermal annealing dramatically increased the mechanical and thermomechanical properties of all materials, and the heat deflection temperature of the biocomposites dramatically increased up to 60 °C with respect to the non-annealed samples. Finally, PNS promoted PLA biodegradation, triggering the swelling of the composites under soil burial, and accelerating the removal of the polymer amorphous phase. These results highlight the potential of combining natural fillers and environmentally benign physicochemical treatments to tailor the properties of PLA biocomposites. The high biofiller content used in this work, in conjunction with the chemical and physico-mechanical treatments applied, increased the thermal, mechanical, and thermomechanical performance of PLA biocomposites while improving their biodegradation behavior. These outcomes allow for widening the application field of PLA biocomposites in those areas requiring a stiff and lightweight material with low deformability and faster biodegradability.

## 1. Introduction

Bioplastics are a potential solution to reduce environmental concerns due to the littering of non-biodegradable plastic wastes [1]. Among bioplastics, poly(lactic acid) (PLA) is regarded as a potential substitute for non-biodegradable plastics since it is fully compostable, and it decomposes releasing non-toxic products [2]. Still, similar to other polymers, it also presents some limits, such as a low crystallization rate and glass transition temperature (T_g_), brittleness, and high cost [3,4,5]. In this regard, the use of additives and fillers can improve the balance between the properties and cost of polymer formulations [6,7]. Indeed, polymer blending with a second solid component for the preparation of composites is a common practice in the compounding of plastic materials. It is often applied in order to improve their mechanical properties, fire behavior, and thermal and electrical performance [7,8]. In addition, it represents a valuable option for reducing the use of plastics and overall costs by replacing a fraction of the polymer matrix with cheap fillers [6,9,10,11,12,13,14].

Pecan nut (*Carya illinoinensis*) has been reported as a biomass suitable for use as a filler in the formulation of biocomposites [11,14,15,16]. In particular, the use of large amounts (up to 50 wt.%) of pecan nutshell (PNS) biowaste as filler in PLA resulted in improved mechanical and rheological properties, as well as a remarkable increase in heat deflection temperature (HDT) promoted by thermal annealing [5,11,14,15,16]. However, poor interaction between this filler and the PLA matrix has been reported [11,15,16]. In this regard, chemical treatments and physical methods that are able to alter the structural properties of biomass are very useful for improving compatibility with the polymer matrix [7,9].

Among these latter, the use of ball milling as a mechanochemical method to reduce biomass particle size and promote morphological and chemical modifications represents an economically and environmentally sustainable alternative to other pretreatment methods [14,17,18,19,20,21]. On the other hand, reactive compatibilization of the matrix is an effective strategy for improving interfacial interactions between polymers and biomass, as the enhancement in stress transfer between components can improve the mechanical properties [9]. This technique is based on introducing functional groups (typically anhydrides, isocyanates, or epoxides) onto non-reactive polymers by using suitable initiators. One of the most used functionalization methods is free-radical grafting, a polymerization-analogous reaction usually initiated by thermal decomposition of thermolabile peroxide groups [22,23,24,25]. Maleic anhydride is the most commonly used monomer for grafting reactions; however, maleic anhydride grafted onto PLA is not yet commercially available due to potentially unfavorable side reactions [26,27,28,29,30,31]. Itaconic anhydride (IA), which is less harmful and very reactive in free radical grafting reactions, represents a potential substitute for the synthesis of biobased grafted PLA [22,23,24,25,27,32].

Based on these considerations, in this paper PLA has been chemically modified with IA (MPLA) and used as a matrix to prepare biocomposites with ball-milled PNS at high charge loading (50 wt.%) to improve filler-matrix interaction and biocomposites’ properties. MPLA-PNS biocomposites were then characterized by means of spectroscopic, thermal, morphological, and mechanical analyses. Additionally, the effect of thermal annealing as a post-processing tool to modify the composite properties was assessed and compared with the results obtained from PLA biocomposites, highlighting the effect of PLA grafting on the HDT. Finally, soil burial tests were conducted to evaluate how the filler affected the biodegradation behavior of the biocomposites.

## 2. Experimental Section

### 2.1. Raw Materials

PLA was NatureWorks Ingeo^TM^ Biopolymer PLA 2003D, 96% L isomer, Melt Flow Rate (210 °C, 2.16 kg) 6 g/10 min, HDT 55 °C (LLC, Blair, NE, USA). All chemical reagents and solvents were obtained from Sigma-Aldrich (Steinheim-Germany). PNS (Asociación Productora de Nuez S.P.R. de R.I, Hermosillo, Mexico) powder (250 μm) was extracted with ethanol at 80 °C (NS1), recovered by filtering on paper, and dried at 60 °C. PNS had an average density of 1.12 g/cm^3^, lignin content of 57 wt.%, and holocellulose content of 39 wt.%.

### 2.2. Ball Milling of the Biomass

NS1 was ball milled in a Retsch PM100 planetary ball milling device (Haan, Germany) using a 125 mL steel milling cup and steel spheres (10 mm diameter) [33]. The spheres/NS1 weight ratio was about 10:1. Ball milling was performed at 650 rpm for 30, 60, and 120 min. As previously reported [14], the average sizes of the milled biomass were 2.7 ± 0.2 μm (30 min), 1.7 ± 0.3 μm (60 min), and 1.5 ± 0.2 μm (120 min).

### 2.3. Modification of PLA via Radical Grafting with Itaconic Anhydride

Grafting of PLA with itaconic anhydride (IA) was performed via radical grafting in one step by reactive-extrusion, using dicumyl peroxide (DCP) as the initiator [22]. IA and DCP (0.5 and 6 wt.%, respectively, of the PLA used) were dissolved in dehydrated acetone (15:1 vol. wt.^−1^), then mechanically mixed with PLA, and dried at 60 °C overnight. The mixture was reactively compounded using a Collin Teach-Line ZK25T co-rotating twin-screw extruder equipped with a pelletizing unit. The following temperature profile was adopted: 145, 165, 180, 165, and 155 °C (from hopper to die), and the screw speed was maintained at 30 rpm. The grafted PLA was ground and purified in methanol using a Soxhlet extractor until the color turned from yellow to white.

#### 2.3.1. Determination of Grafting Degree

Acid-base titration was carried out to assess the degree of grafting. Purified grafted PLA (MPLA) was dissolved in chloroform (1:5 wt. mL^−1^) and the solution was titrated to a phenolphthalein endpoint using potassium hydroxide in methanol (0.04 M) [22]. MPLA was completely soluble and did not precipitate during titration. The degree of grafting was calculated using the following equation:

(1) $$\%\;IA=\frac{N_{KOH}V_{KOH}}{2W_{s}}\times 130.099\frac{g}{\mathrm{mol}}\times 100$$

where *N_KOH_* is the normality (gram equivalent weight of solute per liter of solution) of the *KOH* solution, *V_KOH_* its volume in liters, and *W_s_* is the sample mass (g). An additional validation of grafting reaction occurrence was provided by a postreaction of MPLA with 1-naphthylmethylamine (NMA). MPLA (0.2 g) was dissolved in 20 mL of tetrahydrofuran, and NMA in a molar ratio of 1.2:1 with respect to the anhydride group was added to the solution and stirred at room temperature for 3 h [27]. After the reaction, derivatized MPLA was precipitated by adding 100 mL of methanol. The precipitated product was dried in a vacuum oven at 85 °C overnight. Then, the sample was dissolved in chloroform (0.667 mg mL^−1^) and read at 282 nm using a UV–Vis JASCO V-570 spectrophotometer (JASCO Europe, Cremella, Italy). Neat PLA was also treated with NMA as a control.

#### 2.3.2. Determination of Molecular Weight

Gel permeation chromatography (GPC) was performed with a GPC Max Viscotek equipped with a Malvern TDA with refractive index (RI), right angle laser light scattering (RALS), low angle laser light scattering (LALS), and intrinsic viscosity (IV) detectors. Samples were dissolved and eluted in CHCl_3_ (Romil) at a flux of 0.8 mL min^−1^, with injection volume of 100 μL, concentration of 5 mg mL^−1^, and analyzed through a column set composed of a precolumn and two columns, Phenogel Phenomenex, with exclusion limits 10^6^ and 10^3^ Da. All samples were evaluated with triple point calibration (polystyrene standard Mn = 101.252 kDa and Mw = 104.959 kDa) [34].

### 2.4. Biocomposite Preparation

The formulations at 50 wt.% of charge were coded on the basis of ball milling times of the fillers, MPN1 (0 min), MPN2 (30 min), MPN3 (60 min), and MPN4 (120 min). Biocomposites were compounded using a twin-screw micro-extruder equipped with intermeshing counter-rotating conical screws (HAAKE MiniLab, Thermo Fisher Scientific, Karlsruhe, Germany) following the procedure reported in our previous works [11,14]. Briefly, the temperature adopted was 170 °C and the screw speed was maintained at 50 rpm [11]. Square plates (thickness = 3.0 mm, length = 100 mm) were prepared by compression molding using a Collin P20E platen press (Ebersberg, Germany), at 170 °C (2 min at 0 bar, 1 min at 50 bar, and 2 min at 150 bars) following the conditions reported previously [11,14]. To improve the biocomposites’ thermomechanical properties, the samples were subjected to thermal annealing at 75 °C for 72 h in an oven [35,36,37,38].

### 2.5. Characterization of the Biocomposites

#### 2.5.1. Thermogravimetric Analysis (TGA)

TGA was performed under nitrogen or air atmosphere (flow rate 30 mL min^−1^) using a 7 ± 2 mg sample and a Pyris Diamond TG-DTA analyzer (Perkin Elmer, Waltham, MA, USA). The analysis protocol included a preliminary drying step at 100 °C for 10 min, and a subsequent ramp up to 800 °C at a heating rate of 10 °C min^−1^ [11]. The onset degradation temperature (T_onset_) was evaluated as the temperature corresponding to the 5% weight loss in the TGA curves. The temperature of the maximum degradation rate (T_max_) was calculated as the temperature corresponding to the maximum peak in the derivative thermogravimetric (DTG) plot. Measurements were conducted in replication.

#### 2.5.2. Differential Scanning Calorimetry (DSC)

Calorimetric analyses were performed with a TA DSC-Q2000 instrument under a 50 mL min^−1^ nitrogen flow. Samples (7 ± 3 mg) were first heated from 30 to 180 °C at 5 °C min^−1^, then cooled down to −30 °C at 5 °C min^−1^ and reheated up to 200 °C at 5 °C min^−1^. Glass transition temperature (T_g_), cold crystallization enthalpy and temperature (ΔH_c_, Tc), and melting enthalpy and temperature (ΔH_m_, T_m_) were determined. When a double melting peak was observed, the respective contributions to the melting enthalpy were calculated using the peak analyzer featured in OriginPro 2015 software. Duplicated measurements were carried out.

#### 2.5.3. Heat Deflection Temperature (HDT)

Heat deflection temperature under load was measured by a Thermo Fisher RS6000 (Haake, Germany) rotational rheometer in uniaxial compression mode using parallel plate geometry (plate diameter = 20 mm). The specimens (3 × 10 × 50 mm^3^) were placed on two metal supports (20 mm span length) and loaded flatwise, with midway constant stress of 0.45 MPa and 2 °C/min temperature increase, as is reported by Agustin-Salazar et al. [14]. The HDT was calculated as the temperature at which the specimen deformation was equal to 0.25 mm, which corresponded to a 3% strain. The percent strain (*%D*) was obtained using the following formula:

(2) $$\%D=\frac{\left(d_{0}-d\right)}{d_{0}}\times 100$$

where *d*_0_ is the initial rheometer gap and *d* is the final gap. Duplicate measurements were carried out.

#### 2.5.4. Scanning Electron Microscopy (SEM)

SEM analysis was performed by means of a FEI Quanta 200 FEG scanning electron microscope in high vacuum mode. The observations were performed on sputtered samples with an Au-Pd layer and an acceleration voltage of 20 kV.

#### 2.5.5. Mechanical Tests

The flexural properties of PLA and its biocomposites were determined on 5 bar-shaped specimens with dimensions (3 × 10 × 100) mm^3^ using an Instron model 4505 dynamometer (USA), with a deformation speed of 1 mm min^−1^ and a 48 mm span length, according to ASTM D 638 [11]. For the impact tests, a 3.5 mm V-notch was machined on the same specimens, and the tests were performed using a Ceast M197 Charpy pendulum (Ceast, Turin, Italy) with potential energy equal to 3.5 J and an impact speed of 1 m s^−1^ (ASTM D 256). Ahead of measurement, the specimens were conditioned at 25 °C and 50% relative humidity (RH) for 5 days, and the experimental data are an average of 5 determinations [11].

#### 2.5.6. Soil Burial of Biocomposites

Indoor soil burial experiments were carried out as reported elsewhere [39,40], to simulate natural biocomposite degradation. The test specimens were in the form of bars, 50 mm long, 10 mm wide, and 3 mm thick. The specimens were buried 5 cm deep in commercial garden soil (a mixture of peats and composted vegetal materials) with the following characteristics: pH 6.5, dry apparent density 220 kg m^−3^, total porosity 85% *v/v*. The soil, placed in a pot (60 cm × 40 cm), was allocated in the laboratory at room temperature (23 ± 2 °C) and constant RH of 50%, and kept wet by regular watering. Biodegradation was followed by thermal analysis (DSC and TGA) and sample surface observation by SEM.

## 3. Results and Discussion

### 3.1. Characterization of IA-Grafted PLA

**Figure 1:**
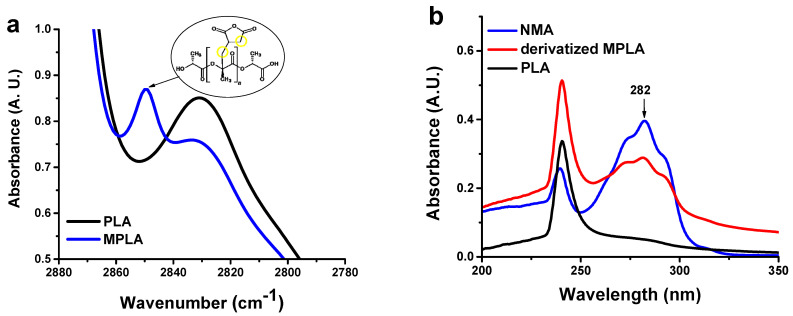
(**a**) FTIR spectra in 2880–2780 cm^−1^ range, and (**b**) UV spectra of neat PLA and derivatized MPLA.

The chemical modification of PLA was assessed by acid-base titration [22,23]. This technique allowed us to calculate an IA content equal to 0.29 ± 0.06 wt.%. PLA modification was first studied through FTIR spectroscopy on thin films, but no difference between plain PLA and MPLA was detected (not shown). This was likely due to the low degree of modification and the overlap of analytical bands in the carbonyl region [27]. FTIR characterization was also conducted on films of high thickness (around 150 μm) (Figure S1a). Compared to the parent polymer, MPLA shows a new weak band around 2850 cm^−1^, which can be assigned to the stretching vibration of CH_2_ functional groups (Figure 1a). [24,32]. However, the presence of CH_2_ moieties in MPLA could also be due to the chain scission phenomena produced by the peroxide initiator on the polyester [32]. Therefore, additional validation of the grafting reaction was provided by a postreaction of MPLA with NMA. The latter contains a chromophore group that can be monitored through UV-vis spectroscopy and also bears an amino functionality that allows its reaction with anhydride groups in the modified polymer [27]. The UV spectra of the derivatized polymer, NMA, and neat PLA are reported in Figure 1b. The absorption relative to the chromophore, with a maximum of 282 nm, is clearly visible in the UV spectrum of derivatized MPLA. This feature further confirmed the presence of IA groups in the modified polymer.

The effects of the radical modification reaction on the molecular weight of the polymer were studied using GPC analysis. The molecular weight data are reported in Table 1, while the superimposed chromatograms of the Refractive Index detector are depicted in Figure S1b. Compared to parent PLA, MPLA showed a reduction of both M_n_ and M_w_, along with an increase in the dispersity, M_w_ M_n_^−1^. These effects could be attributed to radical-promoted degradation accompanying the grafting reaction occurring during the reactive extrusion process, as already pointed out by several authors [27,32,41].

**Figure 2:**
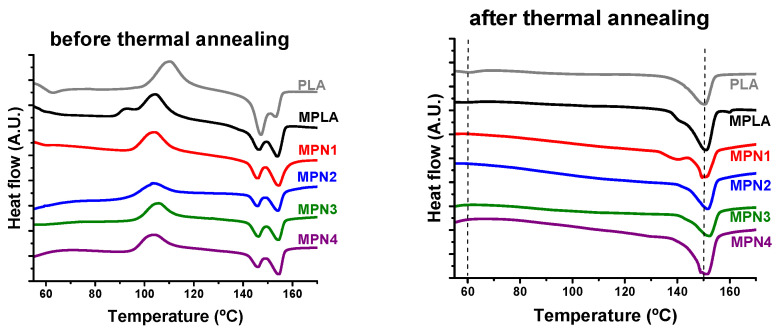
DSC curves during the first heating scan of MPLA and its biocomposites before (**left**) and after (**right**) thermal annealing.

The thermal properties of MPLA were investigated through DSC. The calorimetric curves of the first heating scan are shown in Figure 2, and the corresponding thermal data are reported in Table 2. Both PLA and MPLA exhibit a first signal relative to the glass transition followed by an exotherm, corresponding to cold crystallization and, finally, the melting endotherm. The latter was characterized by peak duplication, as reported in previous papers [14,42]. The experimental data evidenced that after chemical modification T_g_ increased by 5 °C, and the presence of grafted IA also affected the cold crystallization and melting phenomena. Compared to PLA, MPLA showed an increase in both ΔH_c_ and ΔH_m_* along with a decrease in T_c_. Nearly no effect on T_m_ values was detected. The effects recorded could be attributed to a heterogeneous nucleation mechanism. Indeed, even for low grafting degrees, grafted chains or impurities could function as nucleating agents, leading to a higher degree of crystallinity. DSC traces relative to the second heating scan are reported in Figure S2. The relative thermal data (Table S1) show that MPLA evidenced only a slight increase in ΔH_c_ and ΔH_m_* values compared to the parent PLA.

### 3.2. Characterization of MPLA Biocomposites

#### 3.2.1. Thermal Characterization

Figure 2 and Table 2 report the curves and corresponding parameters related to the first heating scan before and after thermal annealing. From the DSC traces depicted in Figure 2, it is observed that all the prepared specimens exhibit a similar behavior characterized by a change in heat capacity due to T_g_ at around 57 °C, followed by an exothermic peak centered at about 104 °C attributed to the cold crystallization phenomenon. At higher temperatures, an endotherm relative to melting is detected. This signal shows two components located at about 145 °C and 156 °C. The DSC parameters reported in Table 2 show that the presence of the filler scarcely affected T_g_ values that remained in the range of neat MPLA. The same holds for T_c_ and T_m_. However, remarkable increases in both ΔH_c_ and ΔH_m_* are noticed. MPLA showed a ΔH_c_ of 28.3 J g^−1^. This value increases to 32 J g^−1^ for MPN1 and remains almost constant independently from the ball-milling treatment. The same trend is noticed for ΔH_m_* that shows a noticeable improvement from the 28.4 J g^−1^ of the neat matrix to the 34.6 J g^−1^ of MPN1. When the filler is submitted to ball milling treatment, this parameter shows a further moderate increase.

After thermal annealing, the first heating scans show no signal related to the glass transition or exothermal peak due to cold crystallization. All the DSC traces show a melting endotherm characterized by a single peak, centered around 150 °C. The latter, in the case of MPLA and MPN1, also display a shoulder on the low temperature side. Calorimetric parameters evidence that the filler scarcely influences melting temperatures. However, ΔH_m_* values indicate that the effect of thermal annealing on melting enthalpy is more remarkable in the presence of the biomass. Neat MPLA exhibits a ΔH_m_* of 32 J g^−1^ while all the investigated biocomposites show higher melting enthalpy values, with a maximum of 43.3 J g^−1^ recorded for MPN4. These results confirm the effectiveness of thermal annealing [14,23,35,43,44,45,46] in promoting the crystallization of MPLA. In addition, they also show that its action can be further improved by the addition of the biomass, which acts as a nucleating agent toward the matrix [14,45]. The higher ΔH_c_ and ΔH_m_* values of the biocomposites compared to the neat MPLA suggest that the filler promoted crystallization by exerting a nucleating action toward the polymeric matrix. Nucleating effects of PNS and similar lignocellulosic biomasses, including sisal fiber and microcrystalline cellulose, have been reported and are related to remarkably improved thermomechanical properties [14,20,45,47,48,49]. For all samples, the melting endotherms appear as a complex signal related to crystal forms of PLA, α-form (ordered) and α′-form [49,50,51]. This behavior was also observed during the second heating scan (Figure S2, and the corresponding thermal parameters shown in Table S1).

The TGA curves of all evaluated samples are reported in Figure S3. The TGA data are shown in Table 3. Interestingly, while under nitrogen, PLA and MPLA showed quite the same T_onset_ (268 °C), in air, MPLA exhibited a value remarkably higher with respect to neat PLA (316 vs. 298 °C). The increased thermal stability in an oxidizing environment is known for PLA and has been attributed to the effect of oxygen-activated crosslinking reactions involving hydrogen abstraction on the tertiary carbon atom of PLA [9]. In this regard, the higher stability of MPLA may be due to unreacted peroxide residues, which can facilitate polymer crosslinking. The biocomposites exhibit T_onset_ values remarkably lower with respect to neat MPLA (316 °C). Particularly, MPN1, MPN2, and MPN3 show T_onset_ around 280 °C while MPN4 exhibits a value of 272 °C. However, nearly no effect of the filler was recorded concerning T_max_. Indeed, the studied biocomposites show values of this parameter ranging between 357 and 362 °C, which are remarkably close or slightly higher with respect to the neat matrix (357 °C). Under a nitrogen atmosphere, the presence of the filler produces an improvement in both T_onset_ and T_max_ compared with neat MPLA. As for the T_onset_ data, biocomposites show values ranging between 273 and 277 °C, which are, on average, higher than the T_onset_ recorded for the neat matrix (268 °C). A more remarkable improvement is recorded for T_max_: in this case, values as high as 328 °C are recorded (MPN3), with a 36 °C increase compared to MPLA. In addition, for this parameter, an effect of mechanochemical treatment was observed. Indeed, samples charged with ball-milled filler (namely MPN2, MPN3, and MPN4) exhibit higher T_max_ values when compared with the biocomposite based on the non-treated filler (MPN1). No discernable dependence of the thermal behavior of the biocomposites on the ball milling time of the filler can be recorded. Char yield is also affected in a remarkable way by the presence of the filler. MPLA shows a residue at 600 °C of 4 wt.%. All the prepared biocomposites exhibit much higher char yields, with values ranging between 21 and 25 wt.%. Similar to T_max_, even in this case, composites based on ball-milled filler evidence a higher charring capability of PNS [14] besides the effect of ball milling on the biomass [52].

#### 3.2.2. Thermomechanical Properties

**Figure 3:**
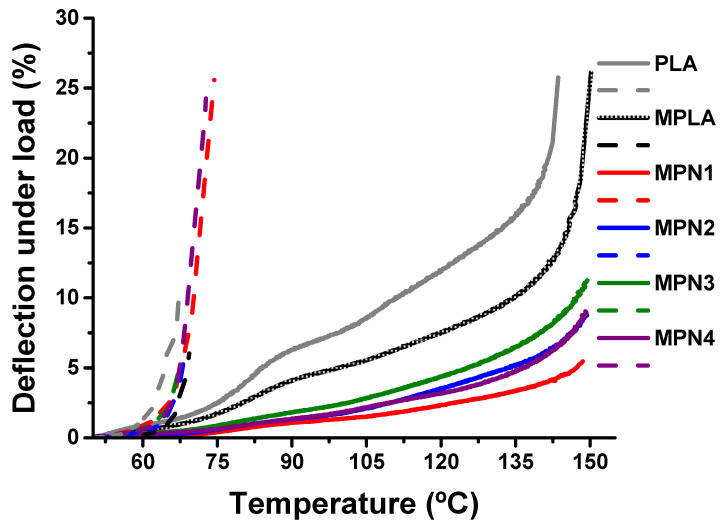
Deflection under load as a function of temperature plots of MPLA and its biocomposites before (dash lines) and after (solid lines) annealing.

HDT determination tests were conducted to establish the influence of filler milling and thermal annealing on the thermomechanical properties of MPLA. The curve relative to deflection under load as a function of temperature before and after annealing is depicted in Figure 3. The HDT values are reported in Table 4. Before annealing, MPLA exhibits higher thermomechanical stability compared to PLA, owing to the larger crystalline fraction developed upon cooling after processing. MPLA and its biocomposites show similar behavior in terms of deformability with temperature. The corresponding HDT values show only a small decrease, ascribable to the presence of the filler. Finally, ball milling does not affect HDT values [14].

After annealing, all the studied samples show a remarkable improvement in thermomechanical behavior, with a noticeable decrease in deformability. The HDT relative to MPLA is 82.9 °C, corresponding to a 15 °C increase with respect to the non-annealed sample. The biocomposites exhibit even larger improvements of HDT, with values as high as 130.7 °C (MPN1). A similar effect of thermal annealing on HDT has been reported both for neat and blended PLA [46,53], as well as for PLA biocomposites [45,54,55] and related to the development of trans-crystallinity at the polymer-matrix interface during annealing [14,45]. The recorded enhancement in thermal resistance allowed evaluation of the strain at high temperatures. Plain MPLA exhibits a strain of 11.7% at 140 °C (to be compared with 18.5% of PLA). Remarkably, the effect of thermal annealing on HDT was outstanding for the biocomposites, also highlighting the beneficial effect of IA grafting on their thermomechanical stability. Indeed, except for MPN3, all MPLA biocomposites show strain values lower than those recorded for analogous biocomposites based on PLA [12]. In particular, the strain value of MPN1 (3.4%) is over 30% lower than that recorded for the corresponding sample PN1 (5.0%) [12], suggesting the potential application of MPLA biocomposites, even in high-temperature applications requiring lightweight and low-deformable material.

#### 3.2.3. SEM Analysis of Cryofractured Surfaces

**Figure 4:**
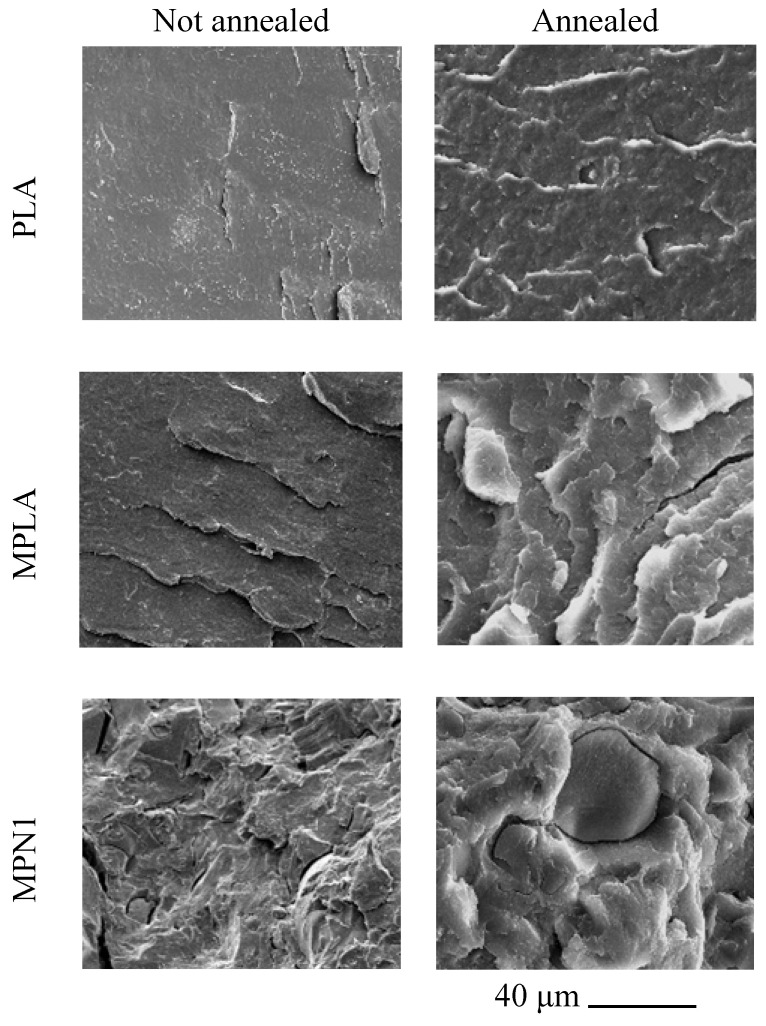
SEM images of cryogenic fracture surfaces of PLA, MPLA, and MPN1 before and after annealing.

Figure 4 shows the micrographs of the cryogenically fractured surfaces of PLA, MPLA, and MPN1 before and after annealing. The respective images for MPN2, MPN3, and MPN4 are reported in Figure S4. In the image relative to the MPN1 sample, the matrix-filler interface reveals some delamination around PNS. Similar results were obtained for the other biocomposites filled with ball-milled biomass (Figure S4). However, in this case, due to a reduction in filler particle sizes, a significant increase in the homogeneity of the sample surface was detected.

Thermal annealing produced an increase in surface roughness. This effect was noticed for both MPLA and biocomposites and is probably related to the higher crystallinity after thermal treatment.

#### 3.2.4. Mechanical Properties

Flexural and impact data of MPLA and its biocomposites, before and after annealing, are reported in Table 5. Flexural data before annealing show that the addition of the biomass produces a moderate improvement of modulus compared to neat MPLA (MPN1 sample). However, the stiffening effect is more limited if ball milling is applied to the charge. All the studied biocomposites, with respect to the plain matrix, exhibit lower stress, and strain at break values. The same embrittlement effect is also noticed for the impact data, which show a drop in resilience.

The mechanical characterization of annealed samples was conducted on plain MPLA and on MPN1, as this latter showed the best performance among the obtained biocomposites. By comparison with the mechanical data of non-annealed specimens, thermal treatment results in a moderate increase in flexural modulus and stress at break for MPLA. At the same time, the strain at break reduces. Conversely, no effect on resilience is recorded. As for the effect of the filler on the properties of the annealed samples, the studied specimens show the same trends recorded before the thermal treatment: MPN1 exhibits a higher flexural modulus and lower properties at break compared with the neat matrix. These outcomes are recorded both in the flexural and the impact mode.

The results relative to MPLA biocomposites reported in this section do not show any significant difference in mechanical performance with respect to the analogous PLA-based system described in a previous work [14]. However, compared to other composites reported in the literature [11,15,16,56,57,58], notwithstanding the large amount of filler introduced into the matrix, only a limited decay of the ultimate properties is observed. The improved interaction between filler and matrix due to chemical modification allows the mechanical properties to be maintained regardless of the high content of biocharge (50%). As for thermal annealing, the enhancement of crystallinity increases the stiffness of the samples, both in flexural and impact testing [14,46,59].

### 3.3. Soil Burial Biodegradation

**Figure 5:**
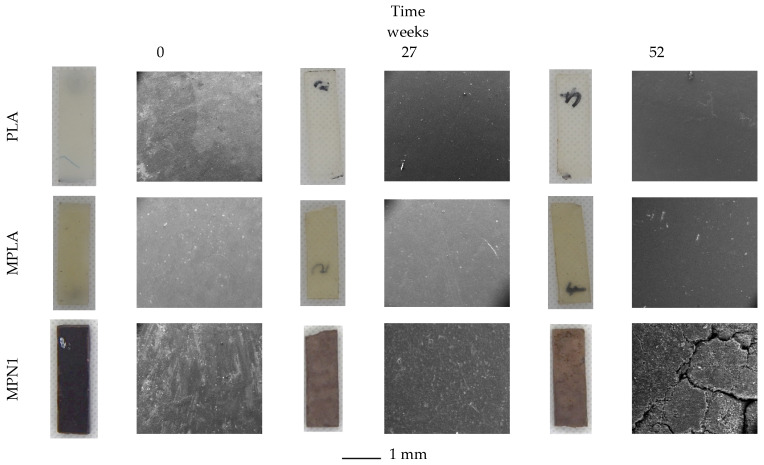
Optical and low magnification SEM images of PLA, MPLA, and MPN1 during biodegradation in soil. The scale bar refers to SEM mages.

**Figure 6:**
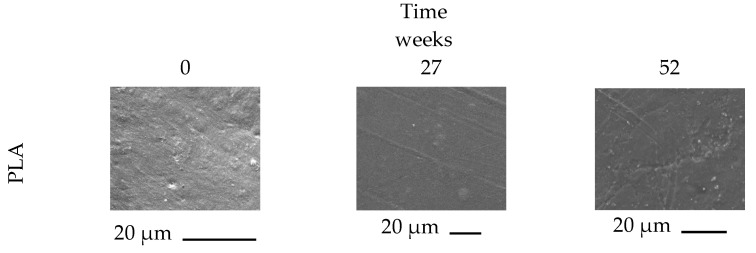

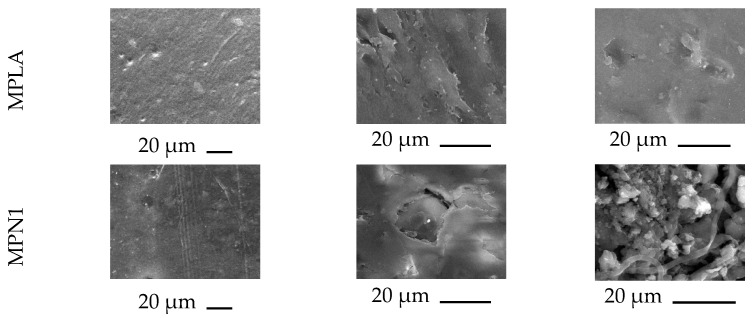
High magnification SEM images of PLA, MPLA, and MPN1 during biodegradation in soil.

All biocomposites (3 mm thick) were subjected to burial in garden soil for up to 52 weeks to obtain insights into their biodegradation behavior. The process was monitored by means of visual analysis, weight measurements, evaluation of thermal properties (TG and DSC analysis), and morphological characterization of the soil-buried samples. Figure 5 shows optical photos of PLA, MPLA, and MPN1. Optical images of the remaining samples are reported in Figure S5. During soil burial, microorganisms can trigger biodegradation, altering the structure of the chemical compounds either through metabolic or enzymatic action [60], and the biodegradation process could be favored by swelling due to water absorption occurring during the test. Indeed, biodegradation in polymeric materials commonly occurs due to: (1) hydrolytic degradation of bonds between monomers, which are then used as energy and carbon sources by soil microorganisms to grow, (2) microbial colonization of the material surface, depolymerization by microorganism extracellular enzymes, and metabolism of the hydrolysis products [61]. Visual examination of the samples demonstrates that after 52 weeks of biodegradation, neither PLA nor MPLA control samples show any change in color, shape, or dimensions (optical images in Figure 5). On the other hand, the tested biocomposites exhibit color fading already after 6 weeks of burying, as evident from the optical images of MPN1. Furthermore, at the end of testing, all composites show a clearly eroded surface (SEM images of Figure 5). A preliminary water sorption test shows no swelling for PLA and MPLA (Figure S6a), while a 4% weight gain after 24 h is noted for all biocomposites, followed by no further weight changes. A similar behavior is recorded during soil burial (Figure S6b), as a single weight gain step is detected for the composites, and except for MPN1, the weight does not change subsequently. This apparently odd behavior was probably due to the biotic colonization of the sample surface (as evidenced by the SEM analysis reported further ahead), which counterbalanced the mass loss due to degradation [62] due to the loss of carbon utilized by the microorganisms to grow [63]. Higher magnification SEM micrographs of the samples after biodegradation are reported in Figure 6 and Figure S7. PLA surface does not show any remarkable feature at both biodegradation times (Figure 6). The same can be said for the MPLA specimen, except for some surface roughness. For the MPN1 composite, at a low biodegradation time (27 weeks), the filler particles surrounded by the polymer matrix start to be visible. A noticeable gap at the interface is clearly noted, while the matrix is characterized by small voids and a few microcracks. The micrograph taken at high biodegradation times also reveals the occurrence of a biofilm on the surface of the material along with erosion of the matrix, which allows the identification of the filler particles. The gaps at the particle–matrix interface are more remarkable, and the cracks on the polymer surface are larger and greater in number. Composites charged with ball-milled fillers show similar biodegradation features. For example, the micrographs relative to the MPN2 sample, depicted in the SI (Figure S7), at low biodegradation times exhibit a relatively smooth surface with the presence of many small cavities. At high biodegradation times, several cracks appear, along with openings attributed to erosion or pulling out of the filler. Compared to MPN1, a finer texture is observed, but this is probably due to the smaller dimensions of the charge. Moreover, surface erosion, swelling, and pull-out phenomena are also observed in all biocomposites. These effects help the breaking and deterioration of the bulk material and, as a consequence, its biodegradation.

The observed soil burial biodegradation effect depends on the specific organisms involved in the processes, which include fungi [64], algae [65], worms [66], snails [67] and even insects [68]. A similar behavior has been reported by Janczak et al. [69] for biodegradation in soil (26 °C) of PLA films (PLA 2003D). In that case, the main changes occurred after 6 months of biodegradation due to fungal activity. It is worth mentioning that the samples analyzed in this work are thicker (3 mm) with respect to those evaluated by Janczak et al. (about 90 mm thick). In this case, the presence of PNS considerably enhanced the biodegradation rate of the materials.

DSC and TGA were performed on buried samples to study the effect of biodegradation on their thermal properties. In Table 6, the data corresponding to TGA are shown, while the related curves are depicted in Figure S3. In an inert atmosphere, nearly no change in thermal behavior due to soil burial is observed for PLA. Conversely, MPLA is more sensitive to biodegradation, as it exhibits an increasing trend of T_onset_ and T_max_ with burial time. These results could be related to coupling reactions occurring over burial time due to the presence of unreacted peroxide or anhydride in the polymer. Before soil burial, all the studied biocomposites showed better thermal performance compared to the plain matrix. However, upon biodegradation, it is not possible to identify a reasonable trend for the reported data. This can be attributed to the complexity of the filler material, along with the different phenomena involved in the biodegradation process.

An important degradation effect was observable as concerns the TGA curves of buried PLA and MPLA, as they turn to be less stable under an air atmosphere than in nitrogen. This finding suggests that the biodegradation process first involves the polymer segments that are responsible for the thermal-oxidative crosslinking of PLA. As far as the biocomposites are concerned, MPN1, and MPN2 showed to be more stable with respect to the rest of the materials, even during the biodegradation test.

**Figure 7:**
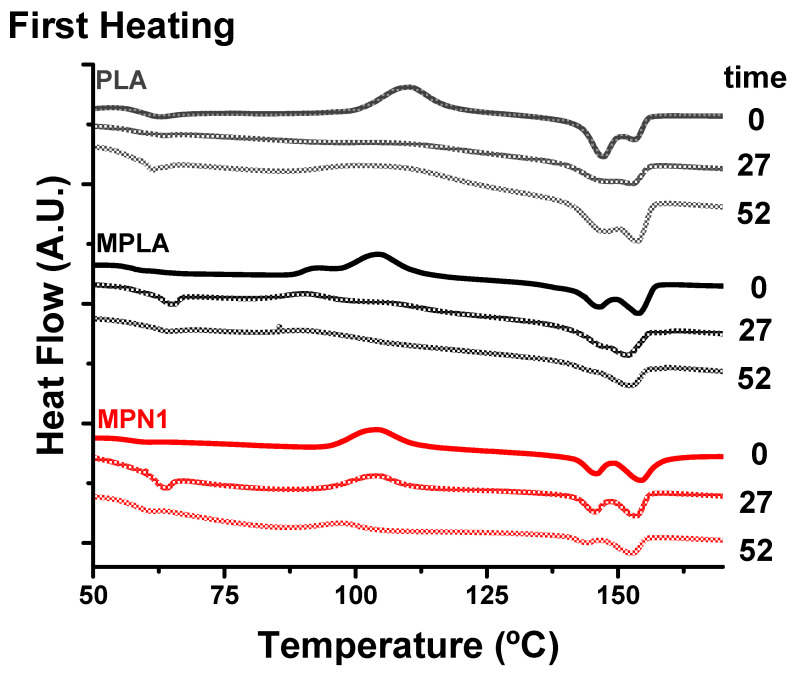
Changes in DSC curves for MPLA biocomposites during biodegradation in soil. The loss of color in the lines corresponds to the increase in biodegradation time.

Figure 7 shows the DSC curves corresponding to the first heating scan of PLA, MPLA, and MPN1. The DSC curves of the remaining biocomposites are presented in Figure S8 in the supplementary material. All the parameters obtained from DSC analysis from first heating scan are shown in Table 7, while the data from second heating scan is reported Table S2. From the DSC traces reported in Figure 7, the most important effect of the biodegradation process is the decrease in cold crystallization. The analysis of the first heating runs shows that after 27 weeks of biodegradation, cold crystallization is completely absent in the PLA trace, very weak in the MPLA plot, and of lower intensity, yet still well detected, in composites. The suppression or decrease in cold crystallization is even more dramatic at high biodegradation times, particularly for biocomposites. Indeed, plots of DSC measurements conducted after 52 weeks of treatment exhibit no cold crystallization phenomena. The biodegradation process mainly involves the amorphous regions of the polymer, which are progressively eroded by the combined action of hydrolysis and microbial biomass, and these phenomena are accelerated by the presence of the biofiller in the composites. On the other hand, all the studied samples show a melting endotherm, whose enthalpy values tend to increase with burial time due to amorphous phase cleavage, while T_m_ remains almost unchanged. This effect suggests that, under the adopted conditions, PLA crystalline chains do not undergo extensive depolymerization under soil burial.

## 4. Conclusions

In this study, PLA was functionalized with IA via a grafting radical process. The obtained MPLA was used for the formulation of biocomposites reinforced with PNS as a filler at a 50 wt.% rate. MPLA showed a low IA content and, compared to the parent polyester, a higher crystallization rate and improved thermo-oxidative stability. The prepared biocomposites showed enhanced crystallizability and better thermal stability with respect to neat MPLA. In addition, no decay of mechanical properties was detected in spite of the high filler content. Thermal annealing affected the morphological and thermomechanical properties of the studied biocomposites by promoting matrix crystallization and producing a noteworthy increase in HDT up to 130 °C. Soil burial tests revealed that the presence of the biofiller in the studied composites favored their biodegradation by increasing water uptake and promoting the development of a biofilm on the sample surface. These effects enhanced the rate and extent of surface erosion. These results highlight that chemical modification of PLA enhances the potential of PNS as a sustainable filler to obtain biocomposites that could be applied for high temperature applications in packaging, including containers, trays, or disposable items.

## Figures and Tables

**Table 1:** GPC data for PLA and MPLA.

	M_n_ kDa	M_w_ kDa	M_w_ M_n_^−1^
PLA	48.1	70.0	1.4
MPLA	42.2	68.9	1.6

**Table 2:** DSC data corresponding to the first heating scan of MPLA and its biocomposites before and after annealing.

	Before Annealing						After Annealing					
	T_g_	T_c_	ΔH_c_	T_m_	ΔH_m_	ΔH_m_ *	T_g_	T_c_	ΔH_c_	T_m_	ΔH_m_	ΔH_m_ *
	°C	°C	J g^−1^	°C	J g^−1^	J g^−1^	°C	°C	J g^−1^	°C	J g^−1^	J g^−1^
PLA	58.8	110.2	23.6	147.0	21.5	24.9	62.8	-	-	-	150.5	28.8
				153.0	3.4							
MPLA	64.1	104.3	28.3	140.9	6.9	28.4	-	-	-	-	150.9	32.0
				153.8	21.7							
MPN1	60.0	104.1	32.2	140.5	14.3	34.6	-	-	-	-	150.3	37.7
				154.3	20.1							
MPN2	62.4	103.3	32.4	145.9	14.8	34.3	-	-	-	-	151.7	41.8
				154.1	19.6							
MPN3	61.0	105.7	37.9	146.1	13.0	36.2	-	-	-	-	152.1	37.8
				154.3	22.0							
MPN4	63.6	103.8	39.4	146.1	12.2	32.4	-	-	-	-	150.9	43.3
				154.5	23.4							

* values were calculated considering 50 wt.% of filler in biocomposites.

**Table 3:** TGA data of MPLA biocomposites under nitrogen and air atmospheres.

	Nitrogen			Air		
	T_onset_ °C	T_max_ °C	Char. Yield * wt.%	T_onset_ °C	T_max_ °C	Char. Yield * wt.%
PLA	268.1	304.6	2.7	297.6	338.6	0.0
MPLA	268.5	292.5	3.9	316.0	356.8	0.0
MPN1	272.9	306.1	20.6	281.0	357.0	3.4
MPN2	277.2	319.9	24.8	280.0	360.0	0.0
MPN3	275.3	327.7	24.1	280.4	362.0	2.8
MPN4	273.6	323.5	24.0	272.3	360.0	1.3

* measured at 600 °C.

**Table 4:** HDT parameters calculated for MPLA and its biocomposites.

	Before	After Annealing	
	HDT °C	HDT °C	Strain * %
PLA	63.1 ± 1.8	77.1 ± 1.5	18.5 ± 1.6
MPLA	67.9 ± 2.1	82.9 ± 1.4	11.7 ± 1.2
MPN1	66.3 ± 1.6	130.7 ± 0.9	3.4 ± 1.1
MPN2	66.8 ± 2.2	115.2 ± 1.6	5.9 ± 0.7
MPN3	66.1 ± 1.2	106.7 ± 1.3	7.6 ± 1.3
MPN4	66.1 ± 1.2	117.6 ± 2.0	5.7 ± 1.4

* measured at 140 °C.

**Table 5:** Mechanical properties of MPLA and its biocomposites.

	FLEXURAL			IMPACT	
	Stress at Break MPa	Modulus MPa	Strain at Break %	Strength N	Resilience KJ m^−2^
Before annealing					
MPLA	78.2 ± 3.8	3646 ± 235	2.6 ± 0.5	103.8 ± 6.7	2.1 ± 0.3
MPN1	61.5 ± 3.3	4824 ± 232	1.2 ± 0.2	98.7 ± 12.7	0.8 ± 0.1
MPN2	52.6 ± 5.8	4562 ± 415	1.1 ± 0.1	95.9 ± 10.4	0.7 ± 0.1
MPN3	48.5 ± 6.4	4053 ± 199	1.2 ± 0.1	82.1 ± 8.1	0.5 ± 0.1
MPN4	48.9 ±6.7	4082 ± 196	1.3 ± 0.1	72.7 ± 13.1	0.6 ± 0.1
After annealing					
MPLA	86.5 ± 3.8	3696 ± 26	1.8 ± 0.2	109.2 ± 24.2	2.1 ± 0.3
MPN1	68.4 ± 3.2	4851 ± 76	1.4 ± 0.1	103.1 ± 19.6	0.9 ± 0.1

**Table 6:** TGA data of MPLA biocomposites at various times of biodegradation in soil under nitrogen and air atmospheres.

	Time Weeks	T_onset_ °C	T_max_ °C	Char. Yield * wt.%	T_onset_ °C	T_max_ °C	Char. Yield * wt.%
Nitrogen					Air		
PLA	0	268.1	304.6	2.7	297.6	338.6	-
	27	269.2	305.6	5.5	261.3	294.4	1.1
	52	272.2	299.4	2.4	265.3	301.0	0.2
MPLA	0	268.5	292.5	3.9	316.0	357.8	0.3
	27	288.4	309.3	0.3	276.0	309.5	9.4
	52	305.2	331.8	0.6	276.9	331.8	6.7
MPN1	0	272.9	306.1	20.6	281.0	356.0	3.4
	27	276.8	319.7	22.5	287.7	311.7	6.6
	52	266.6	301.5	25.9	265.3	296.6	8.2
MPN2	0	277.2	319.9	24.8	280.0	391.3	-
	27	270.8	320.9	20.4	259.2	353.1	3.8
	52	276.8	320.3	19.3	269.9	343.3	-
MPN3	0	275.3	327.7	24.1	280.4	360.0	2.8
	27	253.9	331.3	23.3	254.2	348.6	3.0
	52	258.7	311.9	22.5	254.8	355.3	0.5
MPN4	0	273.6	323.5	24.0	272.3	358.4	1.3
	27	271.7	324	23.9	250.1	305.3	4.7
	52	248.4	303.3	23.7	247.4	333.2	3.2

* measured at 600 °C.

**Table 7:** DSC data corresponding to the first heating scan for MPLA bicomposites during biodegradation in soil.

	Time	Tg	Tc	ΔHc *	Tm	ΔHm *
	Weeks	°C	°C	J g^−1^	°C	J g^−1^
PLA	0	58.8	110.2	23.6	147.0	24.9
					153.0	
	27	63.0	109.7	15.4	152.5	31.6
	52	61.3	107.7	28.1	153.6	35.9
MPLA	0	64.1	104.3	28.3	140.9	28.4
					153.8	
	27	64.7	105.86	24.3	146.5	34.0
					151.67	
	52	63.85	90.75	23.45	144.65	36.92
					152.14	
MPN1	0	60.00	104.14	32.22	140.47	34.64
					154.32	
	27	63.37	104.01	32.20	145.70	39.38
					153.44	
	52	60.28	97.71	8.45	144.20	31.96
					152.44	
MPN2	0	62.43	103.31	32.44	145.86	34.30
					154.08	
	27	62.79	101.42	25.70	145.31	37.94
					152.67	
	52	63.84	113.39	20.10	140.84	65.10
					150.06	
MPN3	0	61.05	105.72	37.9	146.10	36.20
					154.31	
	27	62.38	98.59	20.90	143.12	40.68
					151.85	
	52	62.11	99.15	2.94	142.43	30.24
					151.58	
MPN4	0	63.56	103.81	39.36	146.14	32.40
					154.54	
	27	61.78	99.88	28.58	144.57	45.64
					152.47	
	52	60.37	88.78	1.22	141.55	40.60
					150.58	

* values were calculated considering 50 wt.% of filler in biocomposites.

## Supplementary Materials

The following supporting information can be downloaded at: https://www.mdpi.com/article/10.3390/polym14245532/s1, Figure S1. (**a**) FTIR transmission spectra, and (**b**) Superimposed chromatograms of the Refractive Index detector of PLA and MPLA; Figure S2. DSC curves during second heating scan of MPLA and its biocomposites, before and after annealing; Figure S3. TGA curves (solid) and Derivative TG curve (dashed) for MPLA composites, under air atmosphere and under nitrogen atmosphere; Figure S4. Morphological characterization of PLA and its biocomposites. SEM images of cryogenic fracture surfaces of MPN2, MPN3, and MPN4 before and after annealing; Figure S5. Optical and SEM photos of MPN2, MPN3, and MPN4 biocomposites during biodegradation in soil; Figure S6. Weight change due to (**a**) water absorption, and (**b**) biodegradation in soil of PLA, MPLA and MPLA biocomposites; Figure S7. SEM images of MPN2, MPN3, and MPN4 biocomposites during biodegradation in soil; Table S1. DSC data corresponding to second heating scan for MPLA and its biocomposites, before and after annealing; Figure S8. Changes in DSC curves of MPLA biocomposites during biodegradation in soil; Table S2. DSC data corresponding to second heating scan for MPLA bicomposites during biodegradation in soil.
