# Supplementary material for: Thermomechanical Properties and Biodegradation Behavior of Itaconic Anhydride-Grafted PLA/Pecan Nutshell Biocomposites
Source: Polymers (Basel). 2022 Dec 17;14(24):5532. doi: 10.3390/polym14245532 (PMC9785769; doi:10.3390/polym14245532)
Supplement: Supplementary file 1 [file polymers-14-05532-s001.zip › polymers-2070968-supplementary.pdf]

Supplementary Information

Thermomechanical properties and biodegradation behavior of itaconic anhydride-grafted PLA/pecan nutshell biocomposites

Sarai Agustin-Salazar[1,2,*], Marco Ricciulli[3], Veronica Ambrogi[1,3], Pierfrancesco Cerruti[4,*], Gennaro Scarinzi[1]

[1]Institute for Polymers, Composites and Biomaterials (IPCB-CNR), Via Campi Flegrei 34, 80078 Pozzuoli (Na), Italy
[2]Department of Chemical and Metallurgical Engineering (DIQyM), University of Sonora, Building 5B, Del Conocimiento, Cent ro, C.P. 83000, Hermosillo, Sonora, México
[3]University of Naples Federico II, Department of Chemical, Materials and Production Engineering (DICMAPI), Piazzale Tecchio 80, 80125 Naples, Italy
[4]Institute for Polymers, Composites and Biomaterials (IPCB-CNR), Via Gaetano Previati, 1/E, 23900 Lecco, Italy

*Corresponding authors
Sarai Agustin-Salazar, e-mail: sarai.agustin@ipcb.cnr.it
Pierfrancesco Cerruti, email: pierfrancesco.cerruti@cnr.it

PLA grafting

Functionalization of PLA with IA via radical grafting was performed in one step by reactive-extrusion [1]. In Figure S1a the full FTIR spectrum of PLA and MPLA films (~150 µm of thickness) are presented. A *close up* of the band around 2850 cm^{-1}, assigned to stretching vibration of $-CH_2-$ functional groups, is shown in Fig. 1a. The corresponding discussion is developed in the main text.

In Fig. S1b, the effect on the molecular weight of the polymer due to radical modification reaction is shown in the superimposed chromatograms of the Refractive Index detector. It is possible to observe a shift in the MPLA refractive index curve with respect to retention volume, related to the reduction of both M_n and M_w [2–4].

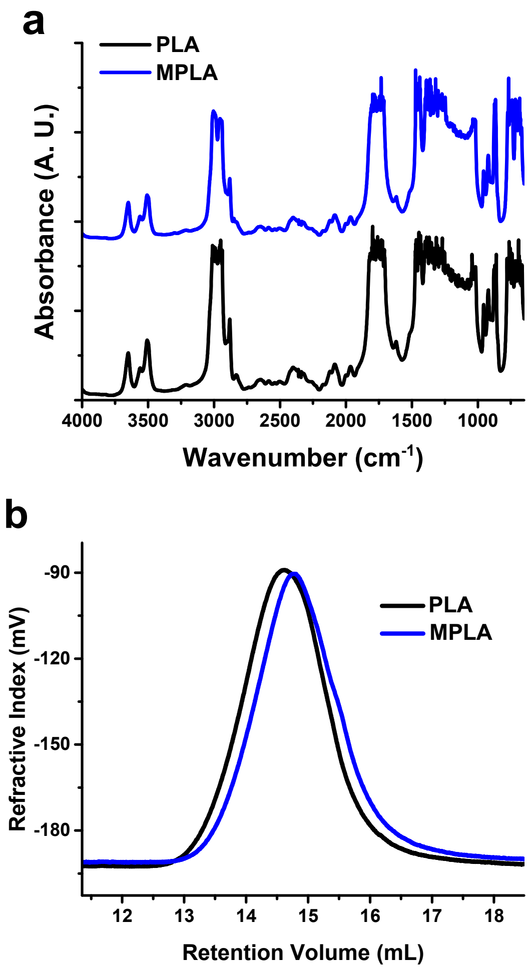

Figure S1. a) FTIR transmission spectra, and b) Superimposed chromatograms of the Refractive Index detector of PLA and MPLA.

Biocomposites characterization

Figure S2 shows the DSC curves, before and after thermal annealing, corresponding to second heating scan. The relative thermal data are shown in Table S1. Before thermal annealing, apart from a slight shift in the thermal events (T_g), not significant differences are noted in MPLA and the biocomposites with respect to PLA. After thermal annealing, a remarkable increase in T_c is noted in PLA. Noticeably, the values of ΔH_c and ΔH_m^* increased in MPLA and its biocomposites. As noted during the first heating scan, the grafting enhanced the crystallization of the polymer, while PNS and thermal annealing further enhanced this effect [5,6,15–17,7–14].

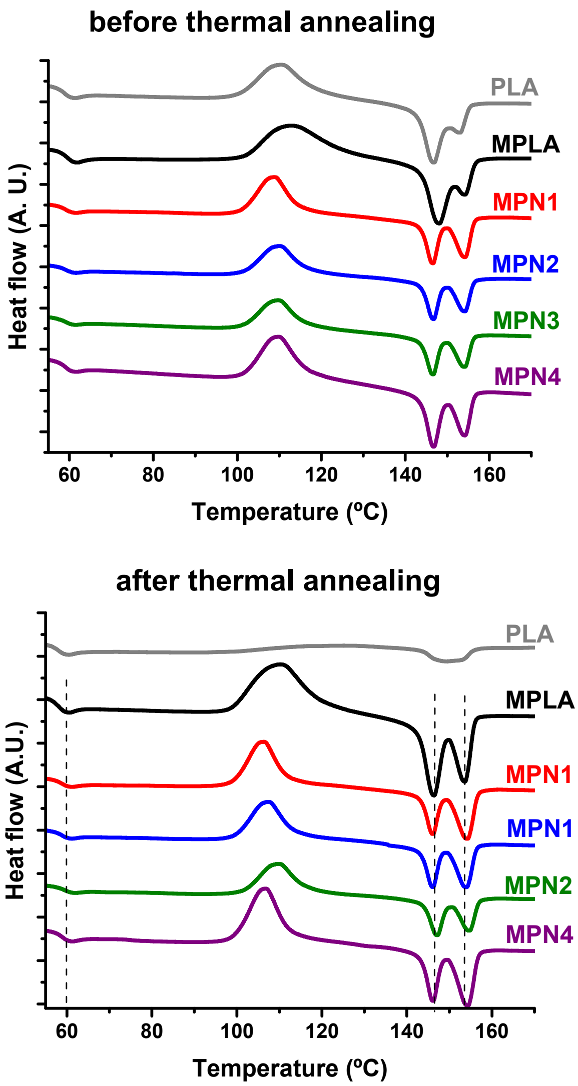

Figure S2. DSC curves during second heating scan of MPLA and its biocomposites, before and after annealing.

Table S1. DSC data corresponding to second heating scan for MPLA and its biocomposites, before and after annealing.

	Before annealing						After annealing				
	Tg<br>ºC	Tc<br>°C	ΔHc<br>J g^{-1}	Tm<br>ºC	ΔHm*<br>J g^{-1}	ΔHm<br>J g^{-1}	Tg<br>ºC	Tc<br>°C	ΔHc<br>J g^{-1}	Tm<br>ºC	ΔHm*<br>J g^{-1}
PLA	58.9	110.6	24.6	146.9<br>153.0	19.5<br>4.6	24.1	57.9	124.0	16.2	149.3	6.5
MPLA	61.5	112.8	27.2	148.1<br>153.6	20.4<br>5.4	25.0	58.2	110.3	32.6	146.3<br>153.4	31.0
MPN1	61.2	106.3	32.0	146.1<br>154.2	18.2<br>9.9	27.70	58.8	106.3	29.3	146.0<br>154.0	29.1
MPN2	61.3	109.9	29.2	146.1<br>153.9	19.6<br>8.7	29.7	58.9	107.4	30.0	146.1<br>154.0	31.1
MPN3	61.1	109.9	31.2	147.0<br>154.7	19.6<br>9.6	25.9	59.4	109.6	24.4	147.1<br>154.6	25.7
MPN4	61.0	109.9	32.0	146.7<br>154.4	20.8<br>8.6	27.02	59.3	106.6	28.4	146.2<br>154.0	29.9

*values were calculated considering 50 wt. % of filler in biocomposites.

TGA curves of all evaluated samples are reported in Figure S3. TGA data in Table 3 and the regarding discussion is reported in the main text of this article. Overall, all the biocomposites exhibited much higher char yields and T_{max} values compared to neat MPLA. This behavior is attributed to the presence of PNS [11] and to ball milling [18].

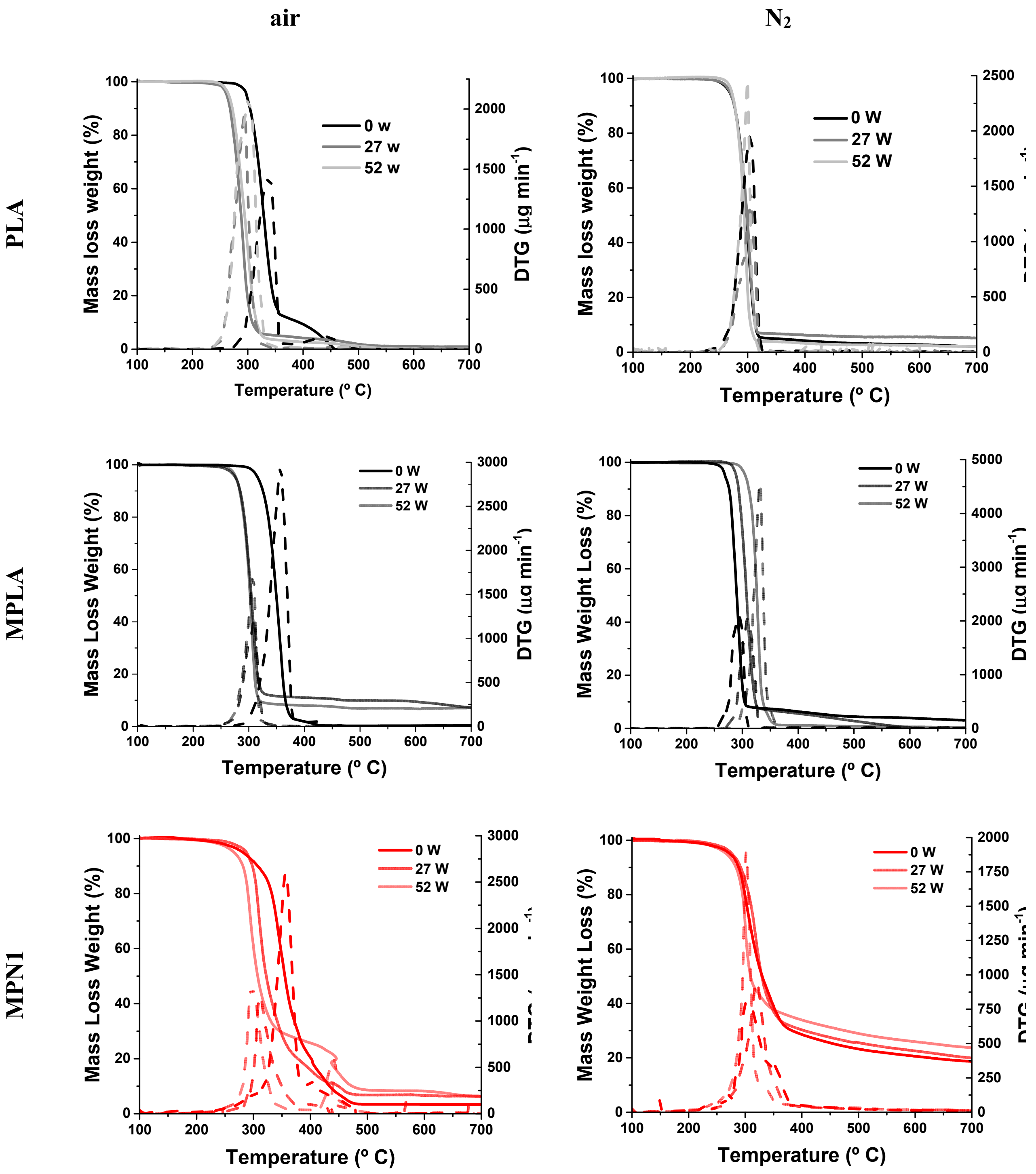

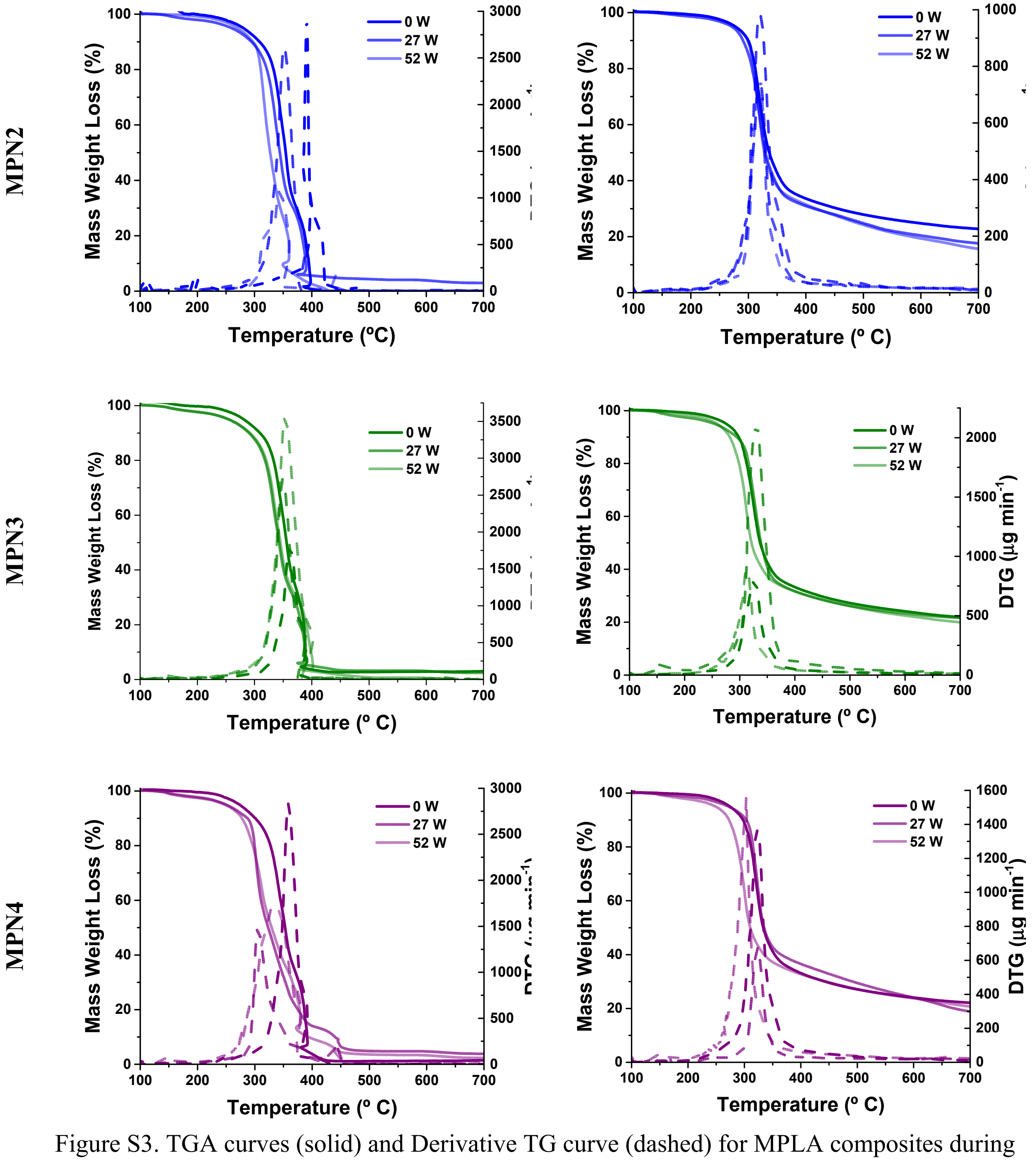

Figure S3. TGA curves (solid) and Derivative TG curve (dashed) for MPLA composites during biodegradation in soil, under air atmosphere and under nitrogen atmosphere.

Figure S4 shows the SEM images for the cryofractured surfaces, before and after annealing, of MPN2, MPN3, and MPN4. Annealed samples show an enhancement in the roughness of surface in MPLA and its biocomposites.

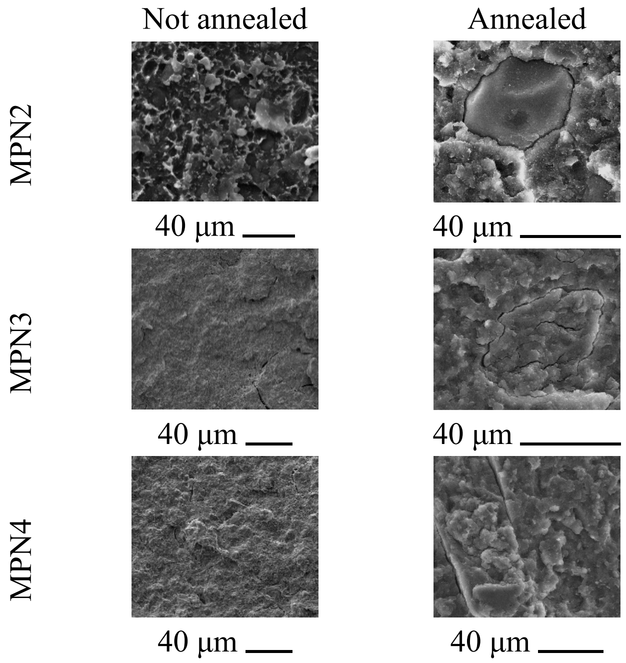

Figure S4. Morphological characterization of PLA and its biocomposites. SEM images of cryogenic fracture surfaces of MPN2, MPN3, and MPN4 before and after annealing.

Soil burial biodegradation

All samples, including plain PLA, were subjected to biodegradation in soil. In Fig. S5, the corresponding optical images and micrographs at high enlargement (50 x) of MPN2, MPN3, and MPN4 are shown. At the end of testing, all composites show a clearly eroded surface. A preliminary water sorption test as well as weight gain curves are reported in Fig. S6. No noticeable changes were observed, probably due to the formation of a surface biofilm which counterbalanced the mass loss due to degradation [19]. In Fig. S7 it is possible to observe this bioactivity on the surface of samples, except for PLA (Fig. 6). Even at low biodegradation times, MPN2 exhibited a smooth surface. This last was more evident as the biodegradation time increased.

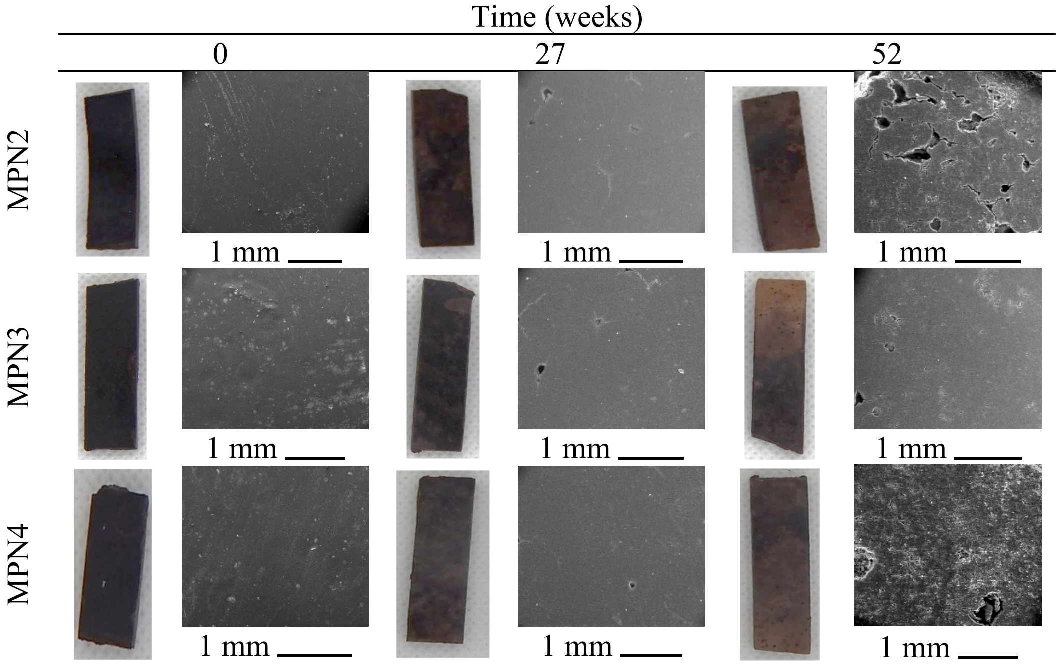

Figure S5. Optical and SEM photos of MPN2, MPN3, and MPN4 biocomposites during biodegradation in soil.

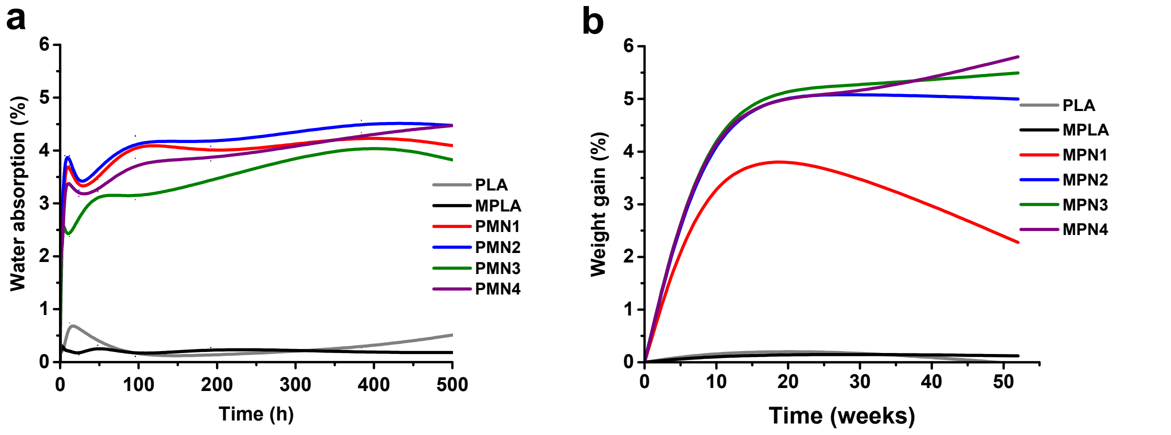

Figure S6. Weight change due to a) water absorption, and b) biodegradation in soil of PLA, MPLA and MPLA biocomposites.

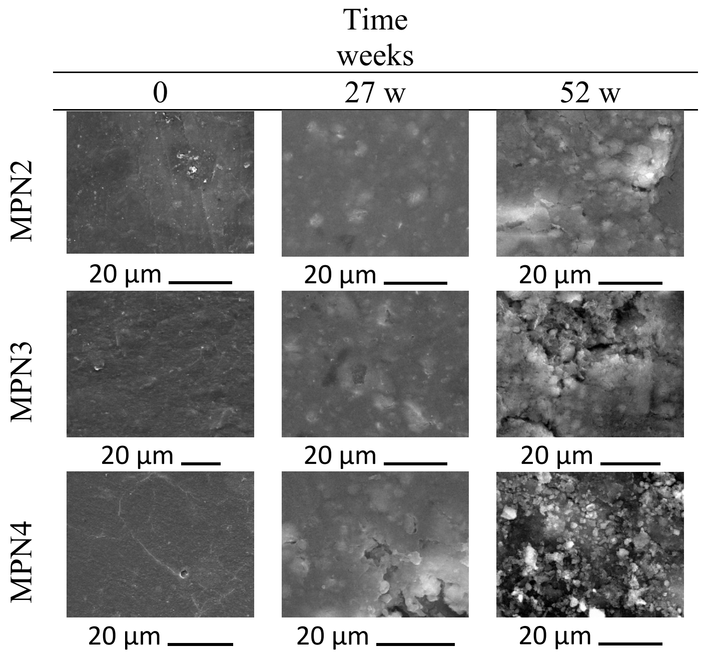

Figure S7. SEM images of MPN2, MPN3, and MPN4 biocomposites during biodegradation in soil.

In Figure S8 and Table S2 the DSC curves and the related parameters obtained during second heating scan for PLA, MPLA, and MPN1, are reported. The first and second heating scans for the remaining biocomposites are also shown in Fig. S8. As well as MPN1, the rest of studied samples show a melting endotherm, whose enthalpy values tend to increase with burial time due to the amorphous phase cleavage, while T_m remains almost unchanged.

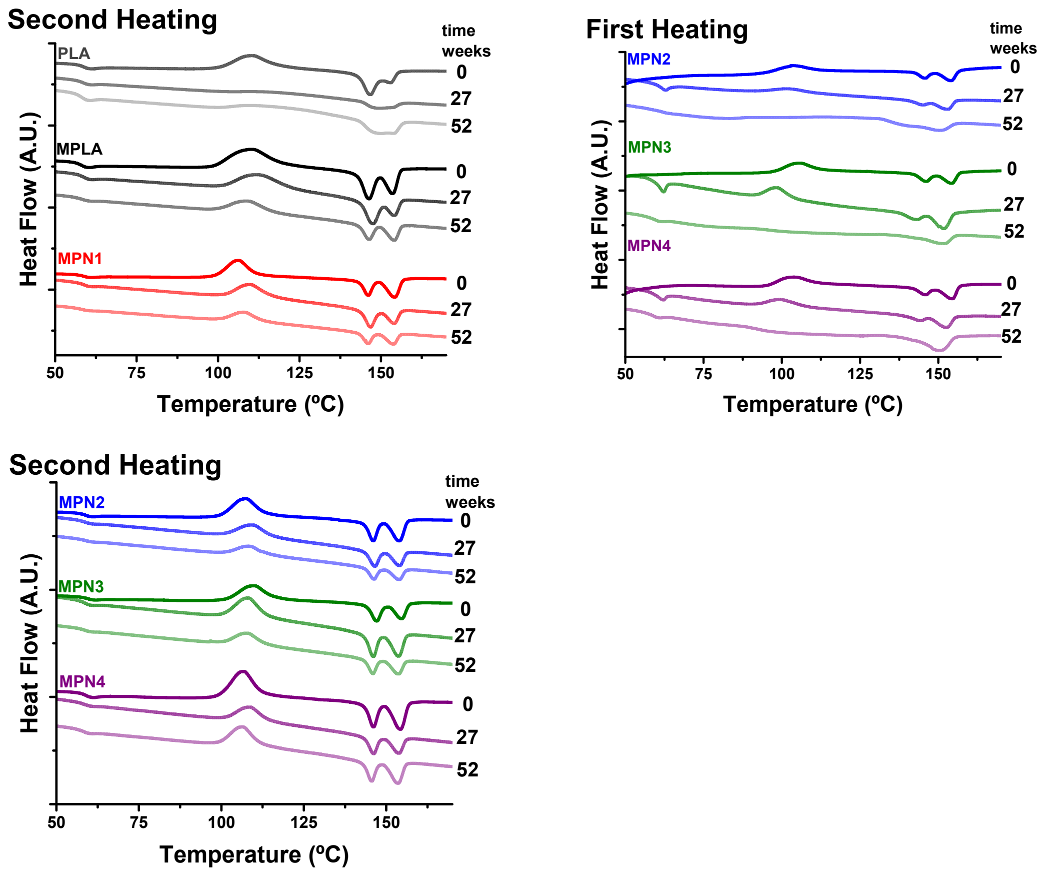

Figure S8. Changes in DSC curves of MPLA biocomposites during biodegradation in soil.

Table S2. DSC data corresponding to second heating scan for MPLA bicomposites during biodegradation in soil.

	time weeks	Tg ºC	Tc °C	ΔHc* J g^{-1}	Tm ºC	ΔHm* J g^{-1}
PLA	0	58.9	110.6	24.6	146.9<br>153.0	24.1
	27	60.3	116.9	8.7	148.9	13.0
	52	60.15	114.1	13.4	149.4	16.7
MPLA	0	61.52	112.84	27.19	148.11<br>153.61	25.04
	27	60.80	112.64	35.29	147.55<br>154.12	33.51
	52	60.51	109.11	38.44	146.29<br>154.09	34.73
MPN1	0	61.25	106.34	32.00	146.13<br>154.17	27.7
	27	60.73	109.76	39.62	146.86<br>154.04	40.26
	52	60.26	107.90	36.62	146.10<br>153.87	37.22
MPN2	0	61.29	109.90	29.22	146.10<br>153.94	29.72
	27	60.89	109.56	35.18	146.55<br>153.99	34.88
	52	60.72	108.69	33.48	146.22<br>154.04	35.12
MPN3	0	61.10	109.90	31.16	147.05<br>154.71	25.96
	27	60.21	107.92	39.36	146.14<br>153.31	32.72
	52	60.29	107.70	40.04	145.96<br>153.51	34.26
MPN4	0	61.05	109.92	31.98	146.67<br>154.38	27.02
	27	60.38	108.91	36.00	146.15<br>153.95	32.90
	52	60.01	106.65	36.67	145.61<br>153.61	33.82

*values were calculated considering 50 wt. % of filler in biocomposites.

References

1. Ku Marsilla, K.I.; Verbeek, C.J.R. Modification of Poly(Lactic Acid) Using Itaconic Anhydride by Reactive Extrusion. *Eur. Polym. J.* **2015**, *67*, 213–223, doi:10.1016/j.eurpolymj.2015.03.054.
2. Liu, W.; Liu, T.; Liu, T.; Liu, T.; Xin, J.; Hiscox, W.C.; Liu, H.; Liu, L.; Zhang, J. Improving Grafting Efficiency of Dicarboxylic Anhydride Monomer on Polylactic Acid by Manipulating Monomer Structure and Using Comonomer and Reducing Agent. *Ind. Eng. Chem. Res.* **2017**, *56*, 3920–3927, doi:10.1021/acs.iecr.6b05051.
3. Rolere, S.; Monge, S.; Rakotonirina, M.D.; Guillaneuf, Y.; Gigmes, D.; Robin, J.J. Chemical Modification of Poly(Lactic Acid) Induced by Thermal Decomposition of N-Acetoxy-Phthalimide during Extrusion. *J. Polym. Sci. Part A Polym. Chem.* **2019**, *57*, 120–129, doi:10.1002/pola.29276.
4. Kučera, F.; Petruš, J.; Matláková, J.; Jančář, J. Itaconic Anhydride Homopolymerization during Radical Grafting of Poly(Lactic Acid) in Melt. *React. Funct. Polym.* **2017**, *116*, 49–56, doi:10.1016/j.reactfunctpolym.2017.05.004.
5. Ku Marsilla, K.I.; Verbeek, C.J.R. Crystallization of Itaconic Anhydride Grafted Poly(Lactic Acid) during Annealing. *J. Appl. Polym. Sci.* **2017**, *134*, 1–11, doi:10.1002/app.44614.
6. Vasanthan, N.; Ly, O. Effect of Microstructure on Hydrolytic Degradation Studies of Poly (l-Lactic Acid) by FTIR Spectroscopy and Differential Scanning Calorimetry. *Polym. Degrad. Stab.* **2009**, *94*, 1364–1372, doi:10.1016/j.polymdegradstab.2009.05.015.
7. Hrabalova, M.; Gregorova, A.; Wimmer, R.; Sedlarik, V.; Machovsky, M.; Mundigler, N. Effect of Wood Flour Loading and Thermal Annealing on Viscoelastic Properties of Poly(Lactic Acid) Composite Films. *J. Appl. Polym. Sci.* **2010**, n/a-n/a, doi:10.1002/app.32509.
8. Orue, A.; Eceiza, A.; Arbelaiz, A. The Effect of Sisal Fiber Surface Treatments, Plasticizer Addition and Annealing Process on the Crystallization and the Thermo-Mechanical Properties of Poly(Lactic Acid) Composites. *Ind. Crops Prod.* **2018**, *118*, 321–333, doi:10.1016/j.indcrop.2018.03.068.
9. Chen, J.; Deng, C.; Hong, R.; Fu, Q.; Zhang, J. Effect of Thermal Annealing on Crystal Structure and Properties of PLLA/PCL Blend. *J. Polym. Res.* **2020**, *27*, 221, doi:10.1007/s10965-020-02206-1.
10. Duan, L.; Zhang, Y.; Yi, H.; Haque, F.; Xu, C.; Wang, S.; Uddin, A. Thermal Annealing Dependent Dielectric Properties and Energetic Disorder in PffBT4T-2OD Based Organic Solar Cells. *Mater. Sci. Semicond. Process.* **2020**, *105*, 104750, doi:10.1016/j.mssp.2019.104750.
11. Agustin-Salazar, S.; Ricciulli, M.; Ambrogi, V.; Cerruti, P.; Scarinzi, G. Effect of Thermal Annealing and Filler Ball-Milling on the Properties of Highly Filled Polylactic Acid/Pecan Nutshell Biocomposites. *Int. J. Biol. Macromol.* **2022**, *200*, 350–361, doi:10.1016/j.ijbiomac.2021.12.101.

12. Běhálek, L.; Maršálková, M.; Lenfeld, P.; Habr, J.; Bobek, J.; Seidl, M. Study Of Crystallization Of Polylactic Acid Composites And Nanocomposites With Natural Fibres By Dsc Method. In Proceedings of the NANOCON 2013. 5th International Conference; Technical University of Liberec: Brno, Czech Republic., 2013; pp. 1–6.

13. Ferry, L.; Dorez, G.; Taguet, A.; Otazaghine, B.; Lopez-Cuesta, J.M. Chemical Modification of Lignin by Phosphorus Molecules to Improve the Fire Behavior of Polybutylene Succinate. *Polym. Degrad. Stab.* **2015**, *113*, 135–143, doi:10.1016/j.polymdegradstab.2014.12.015.

14. Tábi, T.; Hajba, S.; Kovács, J.G. Effect of Crystalline Forms (A′ and α) of Poly(Lactic Acid) on Its Mechanical, Thermo-Mechanical, Heat Deflection Temperature and Creep Properties. *Eur. Polym. J.* **2016**, *82*, 232–243, doi:10.1016/j.eurpolymj.2016.07.024.

15. Zheng, Y.; Fu, Z.; Li, D.; Wu, M. Effects of Ball Milling Processes on the Microstructure and Rheological Properties of Microcrystalline Cellulose as a Sustainable Polymer Additive. *Materials (Basel).* **2018**, *11*, 1057, doi:10.3390/ma11071057.

16. Gracia-Fernández, C.A.; Gómez-Barreiro, S.; López-Beceiro, J.; Naya, S.; Artiaga, R. New Approach to the Double Melting Peak of Poly(l-Lactic Acid) Observed by DSC. *J. Mater. Res.* **2012**, doi:10.1557/jmr.2012.57.

17. Xiao, H.W.; Li, P.; Ren, X.; Jiang, T.; Yeh, J.-T. Isothermal Crystallization Kinetics and Crystal Structure of Poly(Lactic Acid): Effect of Triphenyl Phosphate and Talc. *J. Appl. Polym. Sci.* **2010**, *118*, 3558–3569, doi:10.1002/app.32728.

18. Solikhin, A.; Hadi, Y.S.; Massijaya, M.Y.; Nikmatin, S. Novel Isolation of Empty Fruit Bunch Lignocellulose Nanofibers Using Different Vibration Milling Times-Assisted Multimechanical Stages. *Waste and Biomass Valorization* **2017**, *8*, 2451–2462, doi:10.1007/s12649-016-9765-0.

19. Janczak, K.; Hrynkiewicz, K.; Znajewska, Z.; Dąbrowska, G. Use of Rhizosphere Microorganisms in the Biodegradation of PLA and PET Polymers in Compost Soil. *Int. Biodeterior. Biodegradation* **2018**, *130*, 65–75, doi:10.1016/j.ibiod.2018.03.017.
